# Supplementary material for: Efficacy of ChAdOx1 nCoV-19 (AZD1222) vaccine against SARS-CoV-2 lineages circulating in Brazil
Source: Nat Commun. 2021 Oct 6;12:5861. doi: 10.1038/s41467-021-25982-w (PMC8494913; doi:10.1038/s41467-021-25982-w)
Supplement: Supplementary file 1 — Supplementary Information [file 41467_2021_25982_MOESM1_ESM.pdf]

Efficacy of ChAdOx1 nCoV-19 (AZD1222) vaccine against SARS-CoV-2 lineages circulating in Brazil: Supplementary Tables and Figures

Supplementary Table 1 Hospitalisations (WHO score >=4) by SARS-CoV-2 lineage and age

		ChAdOx1 nCoV-19		Control	
		18-55 years	56 years+	18-55 years	56 years+
Secondary cases (> 21 days after dose 1, < 15 days after dose 2)	Undetermined/no swab	0	0	2	2
	B.1.1.33	0	0	0	1
	P.1 (Gamma)	0	0	1	0
Primary cases >= 15 days after dose 2	Undetermined/no swab	1	0	10	1
	B.1.1.28	0	0	2	2
	P.1 (Gamma)	0	0	1	0
	P.2 (Zeta)	0	0	2	0

Supplementary Table 2 Viral load from swabs (IU/mL)

Lineage	N	Median	Lower Quartile	Upper Quartile	Minimum	Maximum
**All swabs**						
B.1.1.28	78	10421194	3076815	134118446	45720	3719734679
P.2 (Zeta)	234	17495255	1129066	201985761	0	6030847082
P.1 (Gamma)	178	80814649	5753668	526356323	480	18347259144
B.1.1.33	15	1341412	332255	13548956	61164	1408401265
Undetermined	135	117320	1882	63948286	0	3921994942
**Cases included in efficacy analysis**						
B.1.1.28	49	13565118	3433272	239472398	45720	3719734679
P.2 (Zeta)	153	16383823	1129066	208670379	0	6030847082
P.1 (Gamma)	18	28082673	1018799	371165029	98181	1988535214
B.1.1.33	9	4209409	414335	11311270	332255	1408401265
Undetermined	71	2728762	2481	129539823	0	3921994942

Wilcoxon rank sum test comparing cases included in primary efficacy analysis with excluded cases, two-sided p=0.27.
Kruskal-Wallis test comparing viral load across lineages in all participants with swabs, two-sided p=0.0002.

Supplementary Table 3 Days between illness onset and swabbing for NAAT testing

**Lineage**	**N**	**Median**	**Lower Quartile**	**Upper Quartile**
**Known lineage**				
B.1.1.28	76	4	2.5	6
B.1.1.33	15	4	2	6
P.1 (Gamma)	109	4	3	5
P.2 (Zeta)	238	4	3	6
Other B.1 lineage	19	3	2	6
Undetermined	49	8	6	12
Undetermined lineage – Not P.1 (Gamma) or P.2 (Zeta)	79	5	2	6

Supplementary Table 4 Primary symptomatic cases occurring more than 21 days after a single dose of vaccine, by vaccine and lineage

**Lineage**	**ChAdOx1 nCoV-19**	**Control**	**Total**
**B.1.1.28**	0	7	7
**B.1.1.33**	1	2	3
**N.9**	1	0	1
**P.1 (Gamma)**	0	1	1
**P.2 (Zeta)**	6	16	22
**Other B.1 lineage**	1	2	3
**Undetermined**	1	2	3
**Undetermined lineage – Not P.1 (Gamma) or P.2 (Zeta)**	0	6	6

Cases occurred in participants who had received only one dose of vaccine or prior to the receipt of the second dose.

Supplementary Table 5: Oligonucleotide sequences used in this study.

Sequence Name	Target	Purpose	5'>3' sequence	Reaction conc.
2019_nCoV_N1_F	N gene	Quantification	GACCCCAAAATCAGCGAAAT	500 nM
2019_nCoV_N1_R			TCTGGTTACTGCCAGTTGAATCTG	500 nM
2019_nCoV_N1_P			FAM-ACCCCGCATTACGTTTGGTGGACC-BHQ1	125 nM
K417T_FW	S:417	Genotyping	GAGGTGATGAAGTCAGACAAATCGC	600 nM
K417T_RV			GATTGTTAGAATTCCAAGCTATAACGCAGC	600 nM
K417T_P_WT			HEX-TCAGCAATMTTTCCAGTTTGCCCTGG-BHQ1	150 nM
K417T_P_P1			FAM-TCAGCAATMGTTCCAGTTTGCCCTGG-BHQ1	150 nM
L3468V_FW	Orf1a:3468	Genotyping	CACAAGCAGCTGGTACGGACACA	500 nM
L3468V_RV			GCAGAAAGAGGTCCTAGTATGTCAACATGG	500 nM
L3468V_P_WT			HEX-TTAATGTTTTAGCTTGGTTGTACGCTGCTG-BHQ1	125 nM
L3468V_P_P2			FAM-TTAATGTTGTAGCTTGGTTGTACGCTGCTG-BHQ1	125 nM

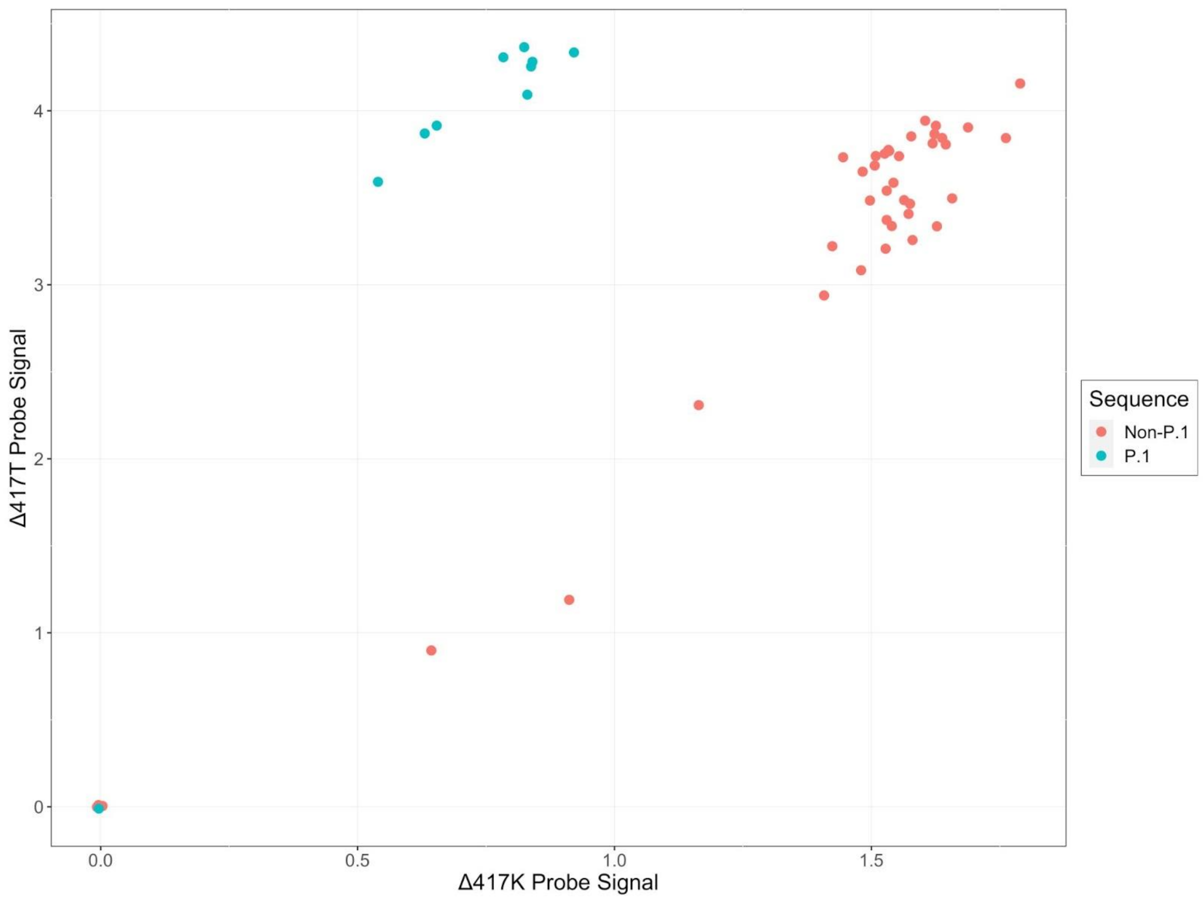

Supplementary Figure 1: Validation of Gamma (P.1) ASP. Allelic discrimination of the Gamma (P.1) assay was validated using 49 sequenced-confirmed samples (10 Gamma (P.1), 39 non-Gamma (P.1)). Assay was performed as described in *methods*; cartesian plot visualizations change in signal for both probes from pre- and post-amplification reads. One Gamma (P.1) sample and two non-Gamma (P.1) samples failed to amplify and are located near the origin of the plot.

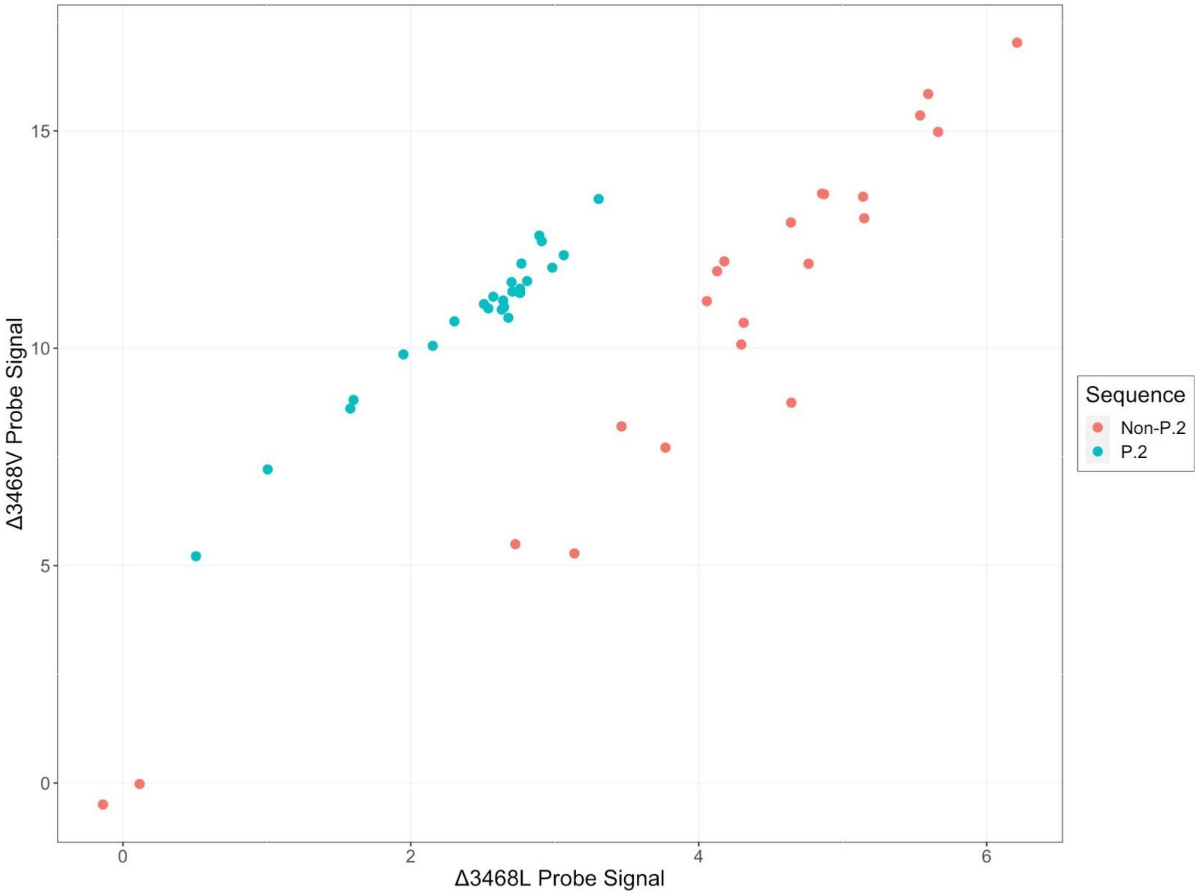

Supplementary Figure 2: Validation of Zeta (P.2) ASP. Allelic discrimination of the Zeta (P.2) assay was validated using 47 sequenced-confirmed samples (25 Zeta (P.2), 22 non-Zeta (P.2)). Assay was performed as described in Methods; cartesian plot visualizations change in signal for both probes from pre- and post-amplification reads. Two non-Zeta (P.2) samples failed to amplify and are located near the origin of the plot.

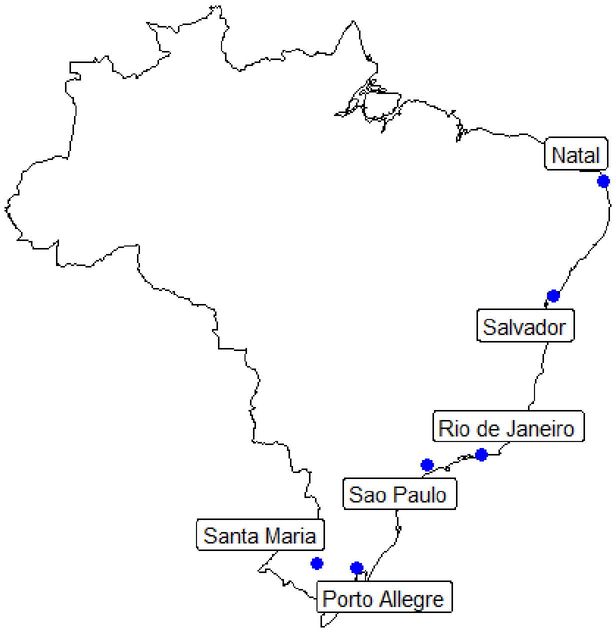

Supplementary Figure 3. Map of Brazil showing location of trial sites collecting samples in the present study.

Supplementary Table 6 List of NAAT assays used in COV003 by study site

Location	Assays
Salvador	1. Seegene Allplex multiplex<br>2. Thermofisher Taqpath multiplex
Porto Alegre	1. US-CDC protocol<br>2. Xpert Xpress SARS-Cov-2, GeneXPert
Rio de Janeiro	1. Allplex, Seegene SARS CoV-2 RT PCR<br>2. Xpert Xpress SARS-Cov-2 GeneXPert<br>3. BioFire Respiratory Panel 2.1 - including SARS-CoV-2, BioMérieux
São Paulo	1. XGEN MASTER COVID-19 kit, Mobius Life Science
Natal	1. Xgen Master COVID-19 Mobius Life<br>2. Norgen Biotek (2019-nCoV TaqMan RT-PCR)
Santa Maria	1. In house assay based on Charite protocol https://www.who.int/docs/default-source/coronaviruse/protocol-v2-1.pdf?sfvrsn=a9ef618c_2 2. Bio-Manguinhos (FIOCRUZ) kit, also based on Charite protocol

Acknowledgements

Clinical Trials Research Governance Office, University of Oxford

Ronja Bahadori

Elaine Chick

Heather House

Claire Riddle

Data and Safety Monitoring Board (DSMB)

George Bouliotis

Steve Black

Elizabeth Bukusi

Cornelia Dekker

Robert Heyderman

Gregory Hussey

Paul Kaye

Bernhards Ogutu

Walter Orenstein

Sonia Ramos

Manish Sadarangani

Department of Paediatrics, University of Oxford

Georg A. Holländer

Endpoint Evaluation Committee

Jeremy Carr

Steve Chambers

Kim Davis

Simon Drysdale

Malick Gibani

Elizabeth Hammershaimb

Michael Harrington

Celina Jin

Seilesh Kadambari

Rama Kandasamy

Toby Maher

Jamilah Meghji

Claire Munro

David Pace

Rekha Rapaka

Robindra Basu Roy

Daniel Silman

Gemma Sinclair

Jing Wang

Jenner Institute, University of Oxford

Iona Tarbet

Nuffield Department of Medicine, University of Oxford

Richard Cornall

Richard Liwicki

Denis Murphy

Elizabeth Salter

Katherine Skinner

Philip Taylor

Oto Velicka

Oxford Research Services (Contracts)

Carly Banner

Sally Pelling-Deeves

Gary Priest

Oxford University Hospitals Trust

Bruno Holthof

Public Affairs Directorate and Divisional Communication Team

Alison Brindle

Alexander Buxton

James Colman

Chris McIntyre

Steve Pritchard
