# Supplementary material for: Efficacy of ChAdOx1 nCoV-19 (AZD1222) vaccine against SARS-CoV-2 lineages circulating in Brazil
Source: Nat Commun. 2021 Oct 6;12:5861. doi: 10.1038/s41467-021-25982-w (PMC8494913; doi:10.1038/s41467-021-25982-w)
Supplement: Supplementary file 3 — Reporting Summary [file 41467_2021_25982_MOESM3_ESM.pdf]

nature research

Corresponding author(s): Dr Merryn Voysey

Last updated by author(s): Aug 17, 2021

Reporting Summary

Nature Research wishes to improve the reproducibility of the work that we publish. This form provides structure for consistency and transparency in reporting. For further information on Nature Research policies, see our Editorial Policies and the Editorial Policy Checklist.

Statistics

For all statistical analyses, confirm that the following items are present in the figure legend, table legend, main text, or Methods section.

n/a	Confirmed	
☐	☒	The exact sample size (*n*) for each experimental group/condition, given as a discrete number and unit of measurement
☐	☒	A statement on whether measurements were taken from distinct samples or whether the same sample was measured repeatedly
☐	☒	The statistical test(s) used AND whether they are one- or two-sided<br>*Only common tests should be described solely by name; describe more complex techniques in the Methods section.*
☐	☒	A description of all covariates tested
☐	☒	A description of any assumptions or corrections, such as tests of normality and adjustment for multiple comparisons
☐	☒	A full description of the statistical parameters including central tendency (e.g. means) or other basic estimates (e.g. regression coefficient) AND variation (e.g. standard deviation) or associated estimates of uncertainty (e.g. confidence intervals)
☐	☒	For null hypothesis testing, the test statistic (e.g. *F*, *t*, *r*) with confidence intervals, effect sizes, degrees of freedom and *P* value noted<br>*Give P values as exact values whenever suitable.*
☒	☐	For Bayesian analysis, information on the choice of priors and Markov chain Monte Carlo settings
☒	☐	For hierarchical and complex designs, identification of the appropriate level for tests and full reporting of outcomes
☐	☒	Estimates of effect sizes (e.g. Cohen's *d*, Pearson's *r*), indicating how they were calculated

Our web collection on statistics for biologists contains articles on many of the points above.

Software and code

Policy information about availability of computer code

Data collection	RedCap 10.6.13 was used for data collection (© 2021 Vanderbilt University)
Data analysis	Data analysis was done using SAS version 9.4 and R version 4.0.4. Bioinformatics analysis was conducted in Python with Pandas 0.25.3. Consensus sequences were aligned using MAFFT version 7.402

For manuscripts utilizing custom algorithms or software that are central to the research but not yet described in published literature, software must be made available to editors and reviewers. We strongly encourage code deposition in a community repository (e.g. GitHub). See the Nature Research guidelines for submitting code & software for further information.

Data

Policy information about availability of data

All manuscripts must include a data availability statement. This statement should provide the following information, where applicable:

- Accession codes, unique identifiers, or web links for publicly available datasets
- A list of figures that have associated raw data
- A description of any restrictions on data availability

Anonymised participant data will be made available when the trials are complete, upon requests directed to the corresponding author. Proposals will be reviewed and approved by the sponsor, investigator, and collaborators on the basis of scientific merit. After approval of a proposal, data can be shared through a secure online platform after signing a data access agreement. All data will be made available for a minimum of 5 years from the end of the trial.

Field-specific reporting

Please select the one below that is the best fit for your research. If you are not sure, read the appropriate sections before making your selection.

☒ Life sciences ☐ Behavioural & social sciences ☐ Ecological, evolutionary & environmental sciences

For a reference copy of the document with all sections, see nature.com/documents/nr-reporting-summary-flat.pdf

Life sciences study design

All studies must disclose on these points even when the disclosure is negative.

Sample size	The data are from a parent randomised controlled trial. The sample size was determined by the number of samples from which a sequence sufficient to define lineage could be generated
Data exclusions	Participants were included in the primary efficacy analyses if they were seronegative to the nucleocapsid protein at baseline, received two doses of vaccine, had follow up for at least 15 days after the second dose, and no prior evidence of infection. Cases were included in the efficacy anlaysis if a lineage was obtained from processing the swab taken for diagnosis, COVID-19 symptoms (in the trial and one or more of: cough, fever great than or equal to 37.8C, shortness of breath, ageusia or anosmia) occurred on day 5 after the second dose or later and before the participant was unblinded as to which vaccines they had received.
Replication	Replication of the analysis was confirmed by independent coding by two statisticians.<br>This study tested the efficacy of a vaccine in participants who were previously unvaccinated and had no prior exposure to SARS-CoV-2. As the population of Brazil now has a large proportion vaccinated, a large proportion of the population exposed to the virus, and substantial viral mutation, replication of overall study results would be difficult to achieve.
Randomization	Participants were randomized with full allocation concealment to receive either ChAdOx nCoV-19 vaccine or MenACWY vaccine.
Blinding	This is a single-blinded trial where participants were blinded.

Reporting for specific materials, systems and methods

We require information from authors about some types of materials, experimental systems and methods used in many studies. Here, indicate whether each material, system or method listed is relevant to your study. If you are not sure if a list item applies to your research, read the appropriate section before selecting a response.

Materials & experimental systems

n/a	Involved in the study	
☒	☐	Antibodies
☒	☐	Eukaryotic cell lines
☒	☐	Palaeontology and archaeology
☒	☐	Animals and other organisms
☐	☒	Human research participants
☐	☒	Clinical data
☒	☐	Dual use research of concern

Methods

n/a	Involved in the study	
☒	☐	ChIP-seq
☒	☐	Flow cytometry
☒	☐	MRI-based neuroimaging

Human research participants

Policy information about studies involving human research participants

Population characteristics	82% of the participants were aged 18-55 years and 70% identified as white. 65% worked in a health or social care setting. 17% had prior cardiovascular disease, 10% had respiratory disease and 5% had diabetes. Median BMI was 26.
Recruitment	Rescruitmen focussed on healthcare workers and others who might be at higher risk of exposure to SARS-CoV-2 to increase study power. The trial population may be healthier than the general population. This does not introduce bias but can affect the interpretation of findings.
Ethics oversight	The trial was conducted according to the principles of the Declaration of Helsinki and was approved by the Brazilian National research Ethics Committee (ref: 32604920.5.0000.5505), and the Oxford Tropical Research Ethics Committee (ref 36-20).<br>The trial is registered at ISRCTN89951424.<br><br>All participants provided informed consent.

Note that full information on the approval of the study protocol must also be provided in the manuscript.

Clinical data

Policy information about clinical studies

All manuscripts should comply with the ICMJEguidelines for publication of clinical research and a completedCONSORT checklist must be included with all submissions.

Clinical trial registration	ISRCTN89951424
Study protocol	https://www.thelancet.com/cms/10.1016/S0140-6736(20)32661-1/attachment/87a97e27-03d4-40c9-8498-7e513e08d265/mmc2.pdf
Data collection	The 6 study sites are are: Sao Paulo, Rio de Janeiro, Salvador, Santa Maria, and Porto Alegre
Outcomes	The primary objective of the trial was to evaluate efficacy of the ChAdox1 nCoV-19 vaccine against NAAT-confirmed COVID-19. The primary symptomatic COVID-19 defined as NAAT+ with at least one of the five COVID symptoms (fever ≥ 37.8oC; cough; shortness of breath; anosmia or ageusia). All NAAT-positive cases occurring before participant unblinding were reviewed by a blinded independent endpoint adjudication committee who assigned severity scores using the WHO clinical progression scale. Only cases adjudicated by the committee as primary outcome cases were included in the analyses. Cases which occurred after unblinding and were not eligible for inclusion in the efficacy analyses were adjudicated by and internal committee
